# The Molecular Epidemiology of Prevalent Klebsiella pneumoniae Strains and Humoral Antibody Responses against Carbapenem-Resistant K. pneumoniae Infections among Pediatric Patients in Shanghai

**DOI:** 10.1128/msphere.00271-22

**Published:** 2022-09-07

**Authors:** Jie Wang, Ruijing Ma, Fen Pan, Yongqin Wu, Yuqing Pan, Yanan Liu, Fangyuan Yu, Jingran Yu, Heyuan Lun, Yingying Shi, Hong Zhang, Ping He

**Affiliations:** a Department of Immunology and Microbiology, Shanghai Jiao Tong University School of Medicine, Shanghai, China; b Department of Clinical Laboratory, Shanghai Children’s Hospital, School of Medicine, Shanghai Jiao Tong University, Shanghai, China; Escola Paulista de Medicina/Universidade Federal de São Paulo

**Keywords:** carbapenem-resistant *Klebsiella pneumoniae*, children, *wzi*-genotyping, O-genotyping, CPS-specific antibody

## Abstract

Carbapenem-resistant Klebsiella pneumoniae (CRKP) has caused wide dissemination among pediatric patients globally and thus has aroused public concern. Here, we investigated the clinical epidemiological characteristics of 140 nonreplicate clinical K. pneumoniae strains isolated from pediatric patients between January and December 2021. Of all isolates, 16.43% (23 of 140) were CRKP strains, which predominantly contained KPC carbapenemase. *wzi* sequencing demonstrated that KL47 (65.22%, 15 of 23) was the most frequent capsular type, followed by KL64 (17.39%, 4 of 23). A total of 23 CRKP strains were classified into three different O-genotypes, including OL101 (65.22%, 15 of 23), O1 (26.09%, 6 of 23), and O3 (8.7%, 2 of 23). Interestingly, KL47 strains were strongly associated with OL101, while KL64 strains were all linked with O1. Some capsule-deficient strains were identified by serological typing, phage-typing, depolymerase-typing, and uronic acid assay. In this study, compared with healthy children, higher titers of anti-capsular polysaccharides (CPS) IgG were first detected in the sera of K47 and K64 K. pneumoniae-infected children, which had the effective bactericidal activity against corresponding serotype K. pneumoniae strains. These findings will facilitate the development of novel therapeutic and vaccine strategies against K. pneumoniae infection in children.

**IMPORTANCE** The emergence of carbapenem-resistant Klebsiella pneumoniae (CRKP) strains resistant to numerous antibiotics and the limited therapeutic options available have become an urgent health threat to the immunocompromised pediatric population. Vaccines and antibodies, especially those targeting capsular polysaccharides, may be novel and effective prevention and treatment options. Thus, it is important to understand the spread of CRKP in pediatric populations. This research presents OL101:KL47 and O1:KL64 as the predominant combinations among CRKP strains in children in Shanghai, China. The primary carbapenemase gene is KPC in CRKP strains. Additionally, this study found elevated levels of anti-CPS IgG against K47 and K64 K. pneumoniae strains in pediatric patients for the first time. The significant bactericidal activity of these anti-CPS IgGs was confirmed.

## INTRODUCTION

Klebsiella pneumoniae can cause various hospital-acquired infections, such as pneumonia, bronchitis, urinary tract infections, and other severe diseases (1). Carbapenem-resistant K. pneumoniae (CRKP), triggering worldwide dissemination, has become an evolving global crisis against human health (2), especially for pediatric patients, causing high morbidity and mortality (3, 4). A report from the CHINET antimicrobial resistance surveillance program between 2005 and 2021 demonstrated that the prevalence of CRKP in China has increased drastically from 2.2% to 24.4%, and the detection rate of CRKP in children was even higher than that in adults (5, 6). Carbapenemase production is the main mechanism contributing to carbapenemic resistance in K. pneumoniae. In China, the predominant carbapenemase expressed in the CRKP strains that infected adults was KPC-2, while in pediatric patients it was NDM-1 (4).

The prevalence of infections caused by hypervirulent K. pneumoniae (hvKP) represents another particular concern. Case reports in recent years have shown that hvKP strains are emerging in the pediatric population, causing life-threatening infectious diseases (1, 7, 8). Several virulence genes, including *peg-344*, *rmpA*, *rmpA2*, *iroB*, and *iucA*, have been identified as being linked closely to hypervirulence. Bioinformatic analyses also showed that these genes are usually present in a large virulence plasmid and are strongly associated with hvKP strains (9).

Serotypes have been widely used to classify Gram-negative species such as Klebsiella isolates. K. pneumoniae can be characterized by capsular K types (at least 77 serotypes) and lipopolysaccharide (LPS) O types (9 serotypes) (10). K-genotyping is now available for determining the capsular types quickly and accurately, based on conservative loci of the *cps* locus, such as *wzi* (10). Likewise, O-genotyping is performed on the main genetic determinant *wb* gene clusters, which contain *wzm* and *wzt* (11). Apart from the methods above, recent studies have shown that bacteriophage typing and depolymerase typing are considered an inexpensive and easy way to type strains as bacteriophages and phage-derived capsule depolymerases exhibiting high specificity for different capsular types (12, 13).

Due to growing antibiotic resistance, novel strategies of controlling CRKP infection are urgently needed. One promising approach to combat antimicrobial-resistant pathogens is inoculating prophylactic vaccines. Recent studies on the polysaccharide protein conjugate vaccine of K. pneumoniae strains suggested that capsular polysaccharides (CPS) could be promising vaccine targets for K. pneumoniae (14). Studies have also shown that anti-CPS monoclonal antibody treatments significantly decreased the dissemination of K. pneumoniae in mice (15, 16), suggesting that it could be another effective strategy against K. pneumoniae infection.

Although research on the molecular epidemiology of K. pneumoniae in adults has been extensively reported, there are only limited data available on the molecular characteristics of these pathogens in the pediatric population. In this study, we investigated the clinical characteristics of K. pneumoniae strains isolated from pediatric patients in Shanghai and further explored its genotypes (O and K typing) and carbapenemase-encoding genes in CRKP strains. Furthermore, the anti-CPS humoral responses in children infected with CRKP strains were detected, and the bactericidal activity of these anti-CPS antibodies was assessed *in vitro*.

## RESULTS

### Clinical characteristics and antimicrobial susceptibility.

From January to December in 2021, a total of 140 nonrepetitive clinical strains of K. pneumoniae were collected from pediatric inpatients. The clinical features were described in Table 1. Of the 140 isolates, 23 (16.43%) strains were classified as CRKP, and 117 (83.57%) were identified as carbapenem-susceptible K. pneumoniae (CSKP) strains. Both cohorts were similar in gender distribution. Among 23 CRKP strains, only one (4.34%) was isolated from neonates; the remaining 22 CRKP strains (95.65%) were isolated from nonneonatal population (Table 1). The proportion of neonates in CRKP groups was significantly lower than in CSKP groups (*P < *0.0001).

**TABLE 1:** Clinical characteristics of the K. pneumoniae strains^*a*^

Characteristics	Total (*n* = 140)	CRKP (*n* = 23)	CSKP (*n* = 117)
Male	75 (53.57%)	13 (56.52%)	62 (52.99%)
Neonate(s)	62 (44.29%)	1 (4.35%)	61 (52.14%)
Nonneonatal patients	78 (55.71%)	22 (95.65%)	56 (47.86%)
Median (range) age of nonneonatal patients (mo)	36 (7 to 96)	72 (48 to 147)	23 (5.5 to 81)
Specimen			
Sputum	86 (61.43%)	11 (47.83%)	75 (64.10%)
Urine	23 (16.43%)	4 (17.39%)	19 (16.24%)
Blood	12 (8.57%)	3 (13.04%)	9 (7.69%)
Others	19 (13.57%)	5 (21.74%)	14 (11.97%)
Isolation wards			
PICUs	22 (15.71%)	8 (34.78%)	14 (11.97%)
NICUs	60 (42.86%)	1 (4.35%)	59 (50.43%)
Pneumology	19 (13.57%)	2 (8.70%)	17 (14.53%)
Hematology	13 (9.29%)	7 (30.43%)	6 (5.13%)
Others	26 (18.57%)	5 (21.74%)	21 (17.95%)
Underlying condition			
Gastrointestinal diseases	33 (23.57%)	11 (47.83%)	22 (18.80%)
Cardiac diseases	65 (46.43%)	3 (13.04%)	62 (52.99%)
Cancers	16 (11.43%)	9 (39.13%)	7 (5.98%)
Cerebral diseases	34 (24.29%)	14 (60.87%)	20 (17.09%)
Premature infants	26 (18.57%)	1 (4.35%)	25 (21.37%)
Invasive procedures	61 (43.57%)	14 (60.87%)	47 (40.17%)
Surgery	24 (17.14%)	6 (26.09%)	18 (15.38%)
Outcome			
Median (ranges) LOS (days)	12 (6 to 30)	36 (24 to 69)	10 (6 to 21)
Mortality	10 (7.14%)	5 (21.74%)	5 (4.27%)

a CRKP, carbapenem-resistant Klebsiella pneumoniae; CSKP, carbapenem-susceptible K. pneumoniae; PICU, pediatric intensive care unit; NICU, neonatal intensive care unit; LOS, length of stay.

Nonneonatal patients who were infected with CRKP had a median age of 72 months (interquartile range [IQR] = 48 to 147 months) (Table 1). As in patients infected by CSKP strains, they had a median age of 23 months (IQR = 5.5 to 81 months) (Table 1). The median age of nonneonatal patients in the CSKP group was significantly younger than that of the CRKP groups (*P = *0.0450).

Most K. pneumoniae strains were derived from respiratory tract specimens (61.43%, 86 of 140), followed by urine (16.43%, 23 of 140) and blood (8.57%, 12 of 140). The specimen distribution between CRKP and CSKP groups was similar (Table 1). The majority of CRKP strains were isolated from pediatric intensive care units (PICU) (34.78%, 8 of 23), while CSKP strains were mainly isolated from neonatal intensive care units (NICU) (50.42%, 59 of 117) (Table 1). The underlying disease of patients between the CRKP and CSKP groups also showed significant differences. The most common underlying conditions of patients infected with CRKP strains were cerebral diseases (60.87%, 14 of 23) (*P* < 0.0001), whereas in the CSKP group, cardiac diseases were most common (52.99%, 62 of 117) (*P = *0.0004). The two groups showed no significant difference in numbers regarding both surgery and invasive procedures (Table 1).

The median length of stay (LOS) of patients infected with CRKP strains was 36 days (IQR = 34 to 69 days), quite a bit longer than the median LOS for patients infected with CSKP strains, which was 10 days (IQR = 6 to 21 days) (*P = *0.0107). In addition, the mortality of children infected by CRKP strains is 21.74% (5 of 23), which is significantly higher than those in CSKP group (4.27%, 5 of 117) (*P = *0.0112) (Table 1).

As is shown in Table S1, all CRKP strains were classified as multidrug-resistant (MDR), and most isolates showed a high level of resistance to β-lactam antibiotics (94.12 to 100%). Nevertheless, the rates of resistance to other tested drugs were much lower than to β-lactam antibiotics. Fortunately, only 26.09% of all strains were resistant to amikacin. Moreover, much lower levels of resistance to colistin (4.35%) and tigecycline (0%) were observed in this study.

10.1128/msphere.00271-22.1 TABLE S1 Antimicrobial activity test of CRKP strains. Download Table S1, DOCX file, 0.02 MB. Copyright © 2022 Wang et al. 2022 Wang et al. https://creativecommons.org/licenses/by/4.0/ This content is distributed under the terms of the Creative Commons Attribution 4.0 International license.

### Resistance genes.

In total, 23 CRKP strains of 140 nonrepetitive clinical strains were successfully identified with the carbapenemase genes. Briefly, *bla*_KPC_ (73.91%, 17 of 23) was the most frequently detected, followed by *bla*_NDM_ (17.39%, 4 of 23), *bla*_IMP_ (4.35%, 1 of 23), and *bla*_OXA-48_ (4.35%, 1 of 23), and one isolate coharboring *bla*_KPC_ and *bla*_OXA-48_ was observed (Table S2). All isolates harbored *bla*_SHV_, followed by other detected β-lactamase genes such as *bla*_CTXM_ (95.65%, 22 of 23) and *bla*_TEM_ (73.91%, 17 of 23) (Table S2). None of the *ampC* genes were observed in these strains (Table S2). The *bla*_KPC_-carrying strains had a resistance rate to amikacin, gentamicin, and sulfamethoxazole/trimethoprim higher than that of *bla*_NDM_-carrying strains (*P* < 0.05) (Table S1).

10.1128/msphere.00271-22.2 TABLE S2 Resistance genes of CRKP strains. Download Table S2, DOCX file, 0.02 MB. Copyright © 2022 Wang et al. 2022 Wang et al. https://creativecommons.org/licenses/by/4.0/ This content is distributed under the terms of the Creative Commons Attribution 4.0 International license.

### *wzi*-genotyping and O-genotyping of K. pneumoniae isolates.

**FIG 1:**
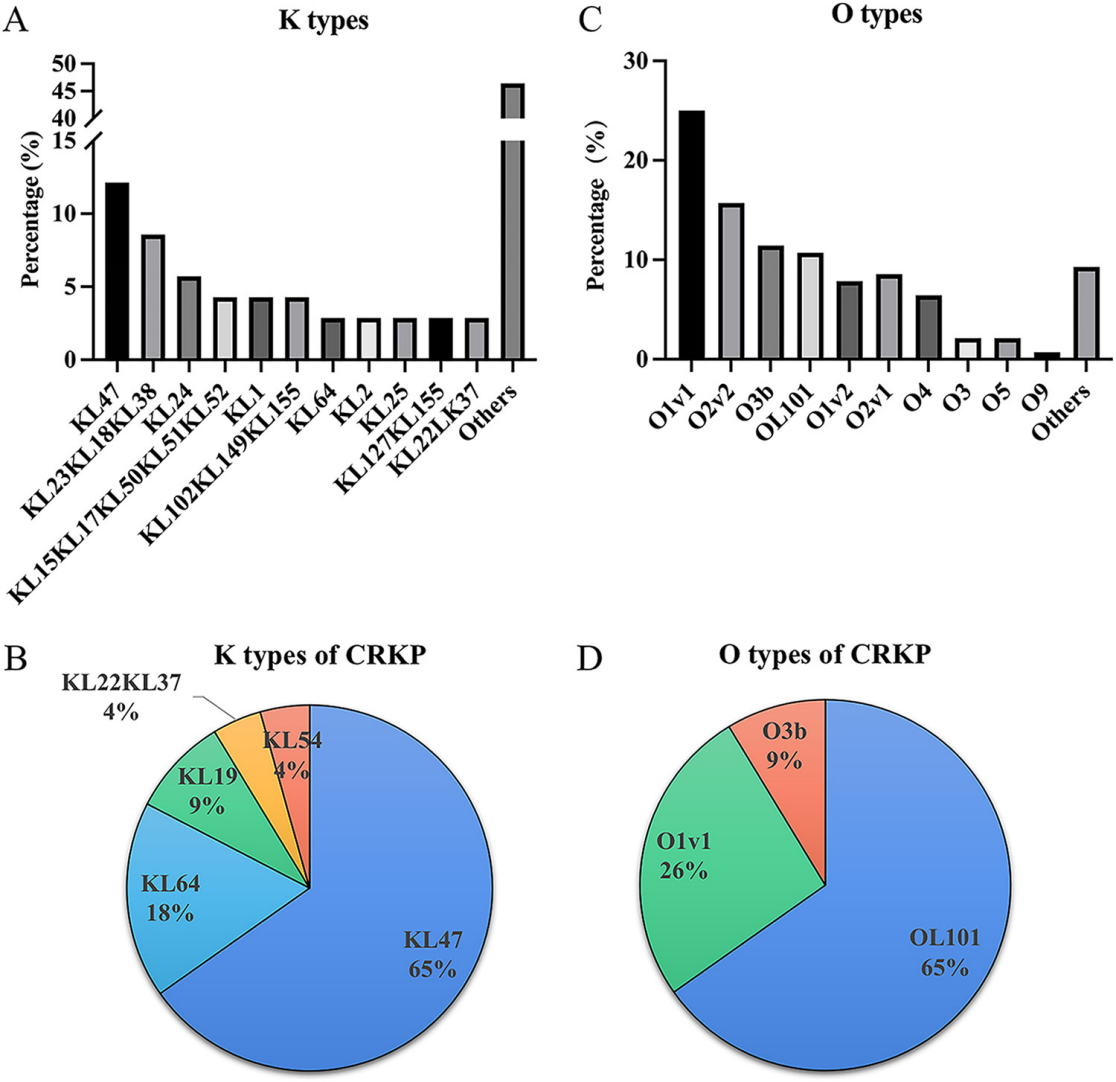
The distribution of K types and O types of isolated K. pneumoniae strains. (A, B) The distribution of K types in a total of 140 strains (A) and carbapenem-resistant K. pneumoniae (CRKP) strains (B). (C, D) The distribution of O-genotypes in a total of 140 strains (C) and CRKP strains (D).

In this study, the total strains achieved K and O typeable ability, up to 98.57% (138 of 140) and 91.43% (128 of 140), respectively. The distribution of all isolates and CRKP strains, which was classified according to K and O types, is shown in Fig. 1. There were 59 distinct K types among the 140 isolates, the most frequent ones being KL47 (12.14%, 17), followed by KL23KL18KL38 (8.57%, 12), and KL24 (5.71%, 8) (Fig. 1A). Twenty-three CRKP strains were assigned to five distinct K types (Fig. 1B). The most frequent capsular type was KL47 (65.22%, 15 of 23) followed by KL64 (17.39%, 4 of 23) (Fig. 1B). KL47 and KL64 strains accounted for more than 80% of CRKP strains, while the remaining capsular types included KL19 (8.70%, 3 of 23), KL22 (4.35%, 1 of 23), and KL54 (1 of 23, 4.35%) (Fig. 1B). There were 10 O-genotypes among the total 140 isolates, including O1v1 (24.29%), O2v2 (15.71%), O3b (11.43%), OL101 (10.71%), O1v2 (8.57%), O2v1 (8.57%), O4 (6.43%), O3 (2.14%), O5 (2.14%), and O9 (0.71%) (Fig. 1C). Twenty-three CRKP strains were classified into three different O-genotypes, including OL101 (65.22%, 15 of 23), O1v1 (26.09%, 6 of 23), and O3b (8.7%, 2 of 23) (Fig. 1D). Interestingly, we found that KL47 CRKP strains were strongly associated with OL101 (OL101:KL47). KL64 CRKP strains were all linked with O1v1 (O1v1:KL64), while a previous study in adults showed that there was intimate connection between KL64 and O2a (17).

### Serological, bacteriophage, and depolymerase typing results of KL47 and KL64 CRKP strains.

We used the traditional serological methods (Quellung assay) and the specific bacteriophage and depolymerase typing methods to further confirm the genotyping results for KL47 and KL64 CRKP strains in this study. Among 15 genotyped KL47 clinical strains, 13 isolates reacted positively with the K47 antisera, and these strains were defined as KL47^+^/K47^+^ strains. The other two strains (strain CRE6 and strain K170) showed negative reactions with the K47 antisera, which defined these two strains as KL47^+^/K47^−^ strains. Similarly, three of four (75%) genotyped KL64 clinical strains were defined as KL64^+^/K64^+^ strains, and one strain (CRE4) was KL64^+^/K64^−^. Previous studies (18, 19) showed that bacteriophages and depolymerases had a high specificity toward the corresponding serotype strains. A clear spot indicated that the phage could infect the bacteria, and a halo showed that the depolymerase could degrade the CPS on the bacterial lawn. All KL47^+^/K47^+^ and KL64^+^/K64^+^ strains were observed to form plaques and halo zones by the spot assay using correlated phage and depolymerase, whereas two KL47^+^/K47^−^ strains and one KL64^+^/K64^−^ strain developed neither plaques nor halos (Table 2). The results were in accordance with the serological methods, which suggested that these strains might have some mutations in CPS-related genes.

**TABLE 2:** Comparison with different methods in capsular typing

Isolate no.	Genotype (*wzi*)	Phages^*a*^		Depolymerase^*b*^	Quellung test
		Plaques	Halos		
CRE10, CRE3, K128	KL64	+	+	+	+
CRE4	KL64	−	−	−	−
CRE5, CRE9, CRE13, CRE14, CRE15, CRE16, CRE18, CRE19, K14, K101, K115, K158, K214	KL47	+	+	+	+
CRE6, K170	KL47	−	−	−	−

a Phage SH-KP152410 is specific for K64 serotype K. pneumoniae strains, and phage SH-KP152226 is specific for K47 serotype K. pneumoniae strains, as previously reported.

b K64-ORF41 is the specific depolymerase for K64 serotype K. pneumoniae strains, and K47-ORF42 is the specific depolymerase for K47 serotype K. pneumoniae strains. The identification of capsular genotypes of all K. pneumoniae isolates were conducted by *wzi* sequencing, and the capsular serotypes of these strains were verified by Quellung test using K64 antisera and K47 antisera (−, negative reaction; +, positive reaction). Phages and depolymerases were tested on corresponding KL type K. pneumoniae strains using the spot method (−, no lysis; +, showed plaques or halos).

### Detection the capsular polysaccharide of KL47 and KL64 CRKP strains by uronic acids assay.

**FIG 2:**
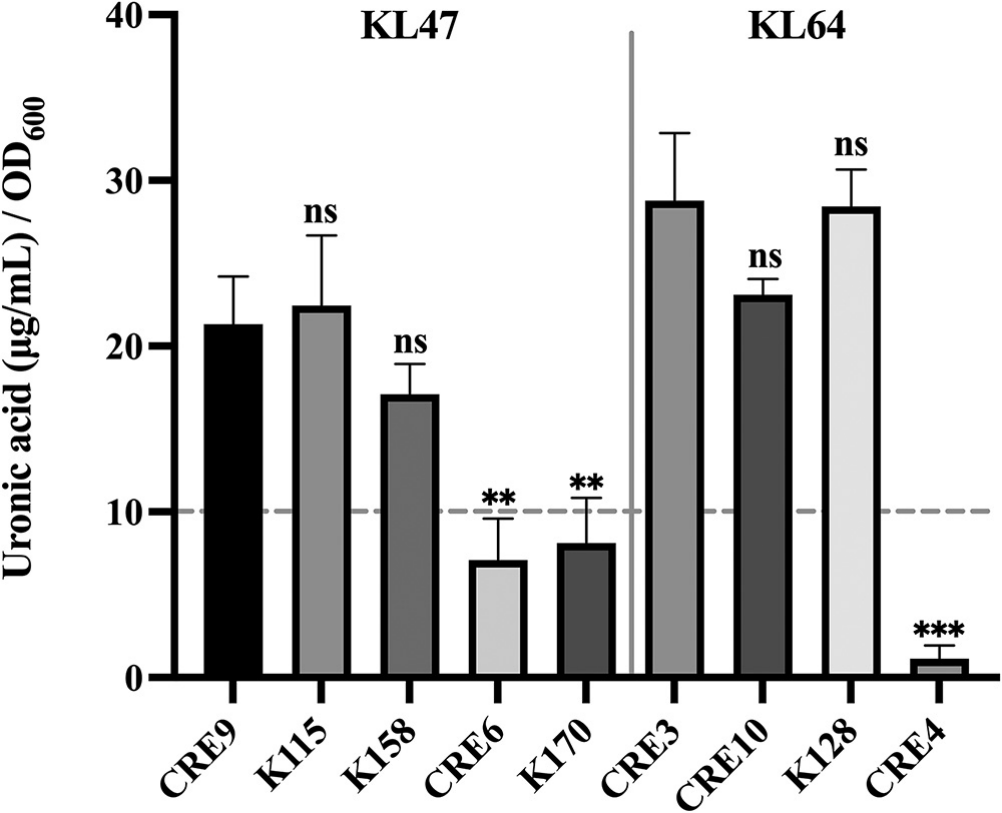
Capsular polysaccharide levels of K. pneumoniae strains measured by uronic acid assay. Three KL47^+^/K47^+^ strains (CRE9, K115, and K158) were randomly selected from CRKP strains and three remaining KL64^+^/K64^+^ strains were CRE3, CRE10, and K128. Two KL47^+^/K47^−^ strains (CRE6 and K170) and one KL64^+^/K64^−^ strain (CRE4) were screened above. The bars represent the standard deviation from the mean of the assay performed in triplicate. The horizontal, dotted gray line at 10 μg/mL was the threshold value to determine the capsular polysaccharides (CPS) mutant strains, as previously reported (44). For the K47 strains, *P* values are displayed above the columns based on the comparison with K. pneumoniae strain CRE9 (unpaired, two-tailed Student’s *t* test). For the K64 strains, *P* values are displayed above the columns based on the comparison with K. pneumoniae strain CRE3 (unpaired, two-tailed Student’s *t* test). Asterisks indicated uronic acid levels that differed significantly between strains, as determined by *t* test. **, *P < *0.01; ***, *P < *0.001; ns, not significant. OD_600_, optical density at 600 nm.

Uronic acids are carboxylated sugars that can be detected in the capsular polysaccharide of K. pneumoniae (20). To quantify the contents of capsular polysaccharide, we measured capsular uronic acids amounts from KL47 and KL64 CRKP strains. The results showed that a significant reduction of uronic acid level was observed in these KL47^+^/K47^−^ strains, compared to those of three randomly selected KL47^+^/K47^+^ strains (Fig. 2). The same results were found with the KL64^+^/K64^−^strain compared with three KL64^+^/K64^+^ strains (Fig. 2). These results verified our supposition that KL47^+^/K47^−^ and KL64^+^/K64^−^ strains were CPS-deficient mutants.

### Anti-CPS antibody responses in clinical patient samples.

**FIG 3:**
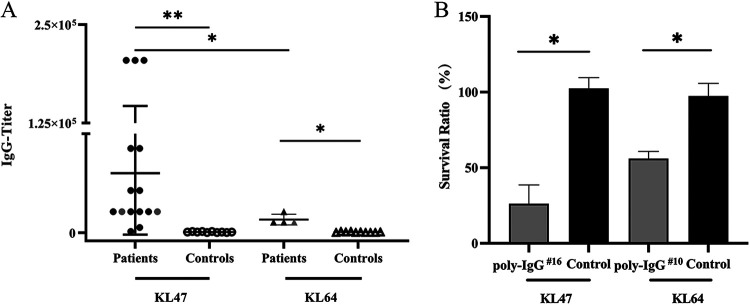
Anti-CPS antibody response and serum bactericidal activity of patients infected with K47 and K64 CRKP strains. (A) Anti-CPS IgG titers detected in patients infected with K47 and K64 CRKP strains versus healthy controls. Each dot on the scatterplot represents an individual’s titer, and the deviations in titers are shown as standard deviations (SD). Gray dots on the scatterplot represent titers of patients infected with capsule-deficient strains (CRE6, K170, and CRE4). Asterisks indicate IgG titers that differed significantly between patients and healthy controls, as determined by two-tailed, unpaired *t* test with Welch’s correction. *, *P < *0.05; **, *P < *0.01. (B) Bactericidal activity of poly-IgG^16^ (serum of the patient infected with K47 K. pneumoniae strain CRE16) and poly-IgG^10^ (serum of the patient infected with K64 K. pneumoniae strain CRE10) against corresponding serotype K. pneumoniae strains were evaluated. K. pneumoniae strain HvKP 4 (K47) or 19-89-1 (K64) was incubated with Hanks’ balanced salt solution (HBSS), baby rabbit complement (or heat-inactivated complement), and heat-inactivated serum of patient (or healthy children) for 1 h at 37°C. The bars indicate SD of the mean. Asterisks indicate the bacteria survival ratio that differed significantly between the patient group and the control group, as determined by two-tailed, unpaired *t* test with Welch’s correction. *, *P < *0.05.

Enzyme-limited immunosorbent assays (ELISAs) were performed to detect the anti-CPS antibodies in the serum of CRKP strain-infected patients. Control serum samples were obtained from healthy children (“noninfected” and “noncolonized”). Compared with healthy children, fifteen patients infected with KL47 K. pneumoniae strains and four patients infected with KL64 K. pneumoniae strains acquired significantly increased IgG levels, in response to K47 CPS and K64 CPS, respectively (*P < *0.001; *P < *0.05) (Fig. 3A). Our results also showed that the IgG titer against K47 CPS was significantly higher than that against K64 CPS (*P < *0.05).

### Patients’ CPS antibodies displayed bactericidal activity against corresponding serotypes of K. pneumoniae strains.

To further explore the immune efficacy of the CPS antibodies against K. pneumoniae, we evaluated the bactericidal activity of poly-IgG^16^ (serum of the patient infected with K47 K. pneumoniae strain CRE16) and poly-IgG^10^ (serum of the patient infected with K64 K. pneumoniae strain CRE10) against the K47 and K64 K. pneumoniae strains, respectively. We found that, compared with the control serum, the antibodies isolated from these two patients had higher bactericidal activity at a 1:10 dilution ratio (Fig. 3B).

### Virulence-associated analysis.

We screened the carriage of five virulence-associated genes, *iucA*, *rmpA*, *rmpA2*, *iroB*, and *peg344* in K. pneumoniae isolates, and the strains with two or more virulence genes were defined as hvKP strains. Among 140 isolates, 11 isolates were identified as hvKP strains. The distribution of hvKP strains was summarized in Fig. S1. The predominant O-genotypes of 11 hvKP isolates were O1 (81.82%, 9 of 11; O1v1 [*n* = 5] and O1v2 [*n* = 4]), followed by O3b (9.09%, 1 of 11) and OL101 (9.09%, 1 of 11). The main capsular serotype was KL1 (54.54%, 6 of 11), followed by KL2 (18.18%, 2 of 11), KL34 (9.09%, 1 of 11), KL25 (9.09%, 1 of 11), and KL47 (9.09%, 1 of 11). KL1 and KL2 were two well known hypervirulent capsule types (16). Moreover, KL1 was highly correlated with O1, containing five hypervirulence associated genes. Among CRKP isolates, one strain (4.3%, 1 of 23) was detected carrying two virulence genes, identified as hypervirulent carbapenem-resistant K. pneumoniae (hv-CRKP).

10.1128/msphere.00271-22.4 FIG S1 The K-types, O-types, and virulence genes distribution of hvKP strains. Download FIG S1, TIF file, 2.9 MB. Copyright © 2022 Wang et al. 2022 Wang et al. https://creativecommons.org/licenses/by/4.0/ This content is distributed under the terms of the Creative Commons Attribution 4.0 International license.

## DISCUSSION

The invasive bacterial infection caused by K. pneumoniae is one of the most common diseases among children. CRKP tops the list of urgent antibiotic-resistant threats recently released by the Centers for Disease Control and Prevention (CDC) (21). Research in recent years has shown that the proportion of CRKP infections in children was higher than that in adults, and there is a rising trend of that in China (6). Given the resistance to carbapenems of CRKP strains and considering that the development and usage of antibiotics upon pediatric patients are relatively finite, novel regimens against CRKP infection are urgently required. Vaccines offer a potentially effective measure against the growing threat of CRKP infection. The CPS of K. pneumoniae facilitates an ideal target for candidate vaccine (22). However, the high diversity of K-antigens makes the design of K. pneumoniae vaccines more challenging. Hence, it is important to monitor the clinically prevalent serotypes of CRKP strains.

Our study described the prevalence of K. pneumoniae isolated from pediatric patients in Shanghai Children’s Hospital and focused on the drug resistance, capsule typing, and anti-CPS IgG response in CRKP-infected patients. Our results showed that the percentages of CRKP isolates in 2021 was 16.43% and had a significant drop in ratio compared to 2016 (34.47%) (4). CRKP is a critical threat to pediatric patients, especially for neonates. A 5-year retrospective study in Turkey observed that CRKP strains were largely isolated from the NICUs (18.1%) and PICUs (39.0%) (23). However, in this study, the proportion of CRKP isolates in the NICU was as low as 1.67% (1 of 60). With the global spread of COVID-19, strict infection prevention measures have been performed in NICUs of Shanghai Children’s Hospital, including active CRE surveillance, single-room isolations, and strict hand sanitation methods, which might have contributed to the dramatic reduction of CRKP infections.

The production of carbapenemase is the major mechanism leading to K. pneumoniae’s resistance to carbapenems (24). The dominant carbapenemase gene in the pediatric patients in China shifted over time. A previous study of CRKP isolates in Beijing Children’s Hospital showed that IMP-4 was the most prevalent type from 2010 to 2012, whereas NDM-1 was reported as the predominant type in 2013 and 2014 (25). Interestingly, our previous research of CRKP isolates in Shanghai Children’s Hospital showed that the most prevalent type in 2016 to 2017 was OXA-232 (4), whereas NDM-1 was the most common in 2013 to 2015 (26), and KPC-2 was supreme during 2018 to 2019 (3, 27). In this study, we revealed that KPC (73.91%,17 of 23) was the prevailing carbapenemase gene in our patient population, which concurred with the prevailing CRKP strains during 2018 to 2019 in Shanghai Children’s Hospital. Unlike the situation in children, CRKP isolates in adults have continued to be the major KPC type in China in recent years (28, 29). The underlying molecular mechanisms that drive the shift of prevalent carbapenemase genes is worthy of further investigation.

To date, surveillance of K. pneumoniae serotypes is important for monitoring the emergence of serotype variations and for advancing strategies in vaccine development. It was more efficient and convenient to deduce K- and O-antigen types using molecular methods, namely, based on the analysis of *cps* locus and *wb* gene cluster, respectively (30, 31).

In this study, the common capsular types were KL47 (65.22%, 15 of 23) and KL64 (17.39%, 4 of 23) among CRKP strains, which accounted for over 80% of all K types, whereas a previous study reported that KL47 (54.7%, 94 of 172) and KL62 (34.9%, 60 of 172) were the major capsular types in 2018 in Shanghai Children’s Hospital (3). The difference of the majority of K types between these years indicated the quick dissemination of high-risk KL64 clones in children. As for K. pneumoniae O-antigens, very limited data were available in pediatric patients. Our results revealed that the most prevalent O-loci in K. pneumoniae strains were O1v1 (25%), followed by O2v2 (15.7%) and O3b (11.4%). One study in the Philippines reported that the most prevalent O-loci were O1v1 (26.6%), O1v2 (13.1%), and O5 (12.0%) from 2015 to 2017 (32). It is noteworthy that in our data, all KL47 CRKP strains were linked with OL101. Moreover, KL64 strains were all identified as O1v1 in our patient population. It was inconsistent with the KL64 strains in adults, which was reported to be closely associated with O2a in China (17). The different correlations between K- and O-antigens in children and adults deserve further investigation.

In recent years, *wzi*-genotyping has been the most commonly used method for epidemiological investigation of K. pneumoniae K types (10, 33). Our previous studies, as well as those of other research groups, found capsule-deficient strains that emerged in clinical K. pneumoniae isolates (19, 34), which could be identified not by *wzi*-genotyping but by depolymerase typing (19). In this study, two KL47 CRKP strains (13.33%, 2 of 15) and one KL64 CRKP strain (25%, 1 of 4) were characterized as capsule-deficient strains, which suggested that nonencapsulated K. pneumoniae strains should be taken into account not only in the design of vaccines but also in the development of anti-CRKP countermeasures.

CPS of K. pneumoniae is a major cell surface antigen that would induce antibodies to protect the host against K. pneumoniae infections (35). Our results showed that significantly higher anti-CPS IgG levels were elicited in patients infected with KL47 and KL64 CRKP strains, which had effective bactericidal activity against respective K type strains. Our results were consistent with a previous study of K. pneumoniae*-*infected adults (36). It was worth noting that the titers of anti-CPS antibodies in sera from patients with capsule-deficient strain infections were still significantly higher than those in control sera. We speculated that these patients were initially infected with encapsulated K. pneumoniae, but the pathogens developed capsule-deficient mutations during disease progression. The evolution and fitness changes of nonencapsulated K. pneumoniae in host deserve further study.

In summary, we characterized 23 CRKP strains among 140 nonreplicate clinical strains of K. pneumoniae in Shanghai Children’s Hospital. KPC was identified as the predominant carbapenemase type in these CRKP strains. OL101:KL47 and O1v1:KL64 were the most prevalent genotype combinations in CRKP strains isolated from children. In addition, we revealed that high-titer anti-CPS IgG levels were elicited in pediatric patients infected with CRKP strains KL47 and KL64. This finding provided important perspectives for developing CPS-based K. pneumoniae vaccines.

## MATERIALS AND METHODS

### Bacterial strains.

A total of 140 consecutive nonreplicate clinical strains of K. pneumoniae were prospectively collected between January and December in 2021 from inpatients in Shanghai Children’s Hospital, an 800-bed tertiary teaching hospital in China. Matrix-assisted laser desorption ionization-time of flight (MALDI-TOF) mass spectrometry (Bruker Diatonic GmbH, Bremen, Germany) was applied to identify strains. To investigate the clinical characteristics of K. pneumoniae strains, the following data were extracted from electronic medical records: the specimen source and patient’s characteristics such as age, gender, wards, underlying diseases, treatment procedures, and outcome.

### Antimicrobial susceptibility testing.

Antimicrobial susceptibility testing was performed by Vitek 2 (bioMérieux, France). The antimicrobials tested were as follows: cefazolin, ceftazidime, ceftriaxone, cefotaxime, cefmetazole, cefepime, ertapenem, imipenem, aztreonam, cefoperazone-sulbactam, ampicillin-sulbactam, piperacillin-tazobactam, ceftazidime-avibactam, amikacin, gentamicin, and nitrofurantoin. The broth microdilution method was used to detect meropenem, colistin, and tigecycline. Antimicrobial susceptibility testing results were unscrambled according to the Clinical and Laboratory Standards Institute (CLSI) guidelines. The activity of tigecycline was interpreted according to the standards of the Food and Drug Administration (FDA), and the MIC breakpoint of colistin was determined based on the standards issued by the European Committee on Antimicrobial Susceptibility Testing (EUCAST). The definition of a CRKP strain was that the strain should be resistant to imipenem, meropenem, or ertapenem. Escherichia coli ATCC 25922 was availed as quality control.

### Resistance gene and virulence-associated gene detection.

We extracted DNA from stored K. pneumoniae strains by the boiling method. Resistance genes include carbapenemase genes (*bla*_KPC_, *bla*_AIM_, *bla*_GIM_, *bla*_SIM_, *bla*_NDM_, *bla*_IMP_, *bla*_VIM_, and *bla*_OXA-48_), ESBL genes (*bla*_CTX-M_, *bla*_TEM_, and *bla*_SHV_), and *ampC* genes (*MOX*, *FOX*, *DHA*, *CIT*, *AAC*, and *EBC*). All MDR K. pneumoniae strains were identified by polymerase chain reactions (PCR) with specific primers as previously described (Table S3) (3, 37). MDR was defined as the resistance to three or more different antimicrobial classes (38). Five hypervirulence-associated factors, including *peg-344*, *rmpA*, *rmpA2*, *iroB*, and *iucA* were screened by PCR with specific primers as previously described (Table S3) (9).

10.1128/msphere.00271-22.3 TABLE S3 List of primers. Download Table S3, DOCX file, 0.03 MB. Copyright © 2022 Wang et al. 2022 Wang et al. https://creativecommons.org/licenses/by/4.0/ This content is distributed under the terms of the Creative Commons Attribution 4.0 International license.

### *wzi*-genotyping of K-antigens.

The capsular genotypes of 140 isolates were determined by *wzi* sequencing according to the method described by Brisse et al. (30). The primers of *wzi* are listed in Table S3. Capsular polysaccharide *wzi* alleles were sequenced, and the K types were analyzed using the K. pneumoniae sequence typing database (https://bigsdb.pasteur.fr/) (39). K types based on the K locus arrangement is called the KL series (19).

### Genotyping of O-antigens.

The method of O typing was described previously (10, 31). Briefly, we exploited the specific primers targeting O1/O2, O3, O4, O5, O8, O9, and O12 alleles to determine preliminary O-antigens, which was performed by PCR. In addition, the primers targeting the O1 and O2 *wbbY* alleles were utilized to differentiate O1 and O2. O1/O2 strains were then typed for variants 1 and 2 by PCR using primers (*wbbO*-fw and *hisI*-rev) as previously published (40). The primers used to determine the different O3 subtypes were performed as previously described (41). For those O-antigens that could not be identified, a previous study that designed novel *rfb* loci sequences as OL[n] was referenced and optimized by us to further type O-antigens (42). We designed two sets of PCR primer pairs to determine OL101 genotype based on *wzm* and *wzt* genes, according to DNA sequencing data LT174596.1 (42). All primers are listed in Table S3.

### Serological K typing of KL47 and KL64 strains.

Traditional serological methods, namely, the Quellung reaction (43), were used to further confirm the serotyping results for KL64 and KL47 clinical strains. The K47 antisera and K64 antisera were prepared as described previously (18, 19) and were applied for serological typing methods. The Quellung reactions were conducted with a protocol described previously (19). Strains were cultured in LB broth at 37°C overnight followed by centrifugation and were then resuspended in phosphate-buffered saline (PBS). A drop of suspension was then mixed with K64 antisera or K47 antisera and methylene blue solution on a glass slide. After 15 min of reaction, the reactants were observed under the optical microscope for the Quellung reaction.

### Phage and depolymerase typing of KL47 and KL64 strains.

Spot assay was performed to determine K47 and K64 types using phage and phage-derived polysaccharide depolymerases (13). Phage SH-KP152410 and depolymerase K64-ORF41 were specific for K64 K. pneumoniae strains (19), and phage SH-KP152226 and depolymerase K47-ORF42 were specific for K47 K. pneumoniae strains (18). Seropositive strains were further determined by the development of plaques (by phage) or the formation of translucent halo zones (by depolymerase).

### Purification of CPS and uronic acid quantification.

The CPS of K. pneumoniae strains was purified based on previously described protocols (44). Overnight cultures of K. pneumoniae strains (1 × 10^9^ CFU) were centrifuged, resuspended in 500 μL PBS, and then mixed with 200 μL 1% Zwittergent in 100 mM citric acid buffer at 55°C for 30 min. Bacterial cells were centrifuged at 10,000 × *g* for 10 min; then 0.4 mL of the cell-free supernatant was mixed with 1.6 mL absolute ethanol and incubated for 30 min at 4°C. After being centrifuged, the samples were rehydrated with 125 μL of water overnight and then were mixed with 0.5 mL of 12.5 mM sodium tetraborate in concentrated sulfuric acid (45). A standard curve of glucuronic acid was utilized to calculate uronic acid concentrations (46).

### Detection of anti-CPS antibodies in serum by CPS ELISA.

ELISAs described previously (36, 47) were employed to detect the levels of CPS antibodies in the serum of patients. Briefly, each well of a 96-well plate was coated overnight with 100 μL of polysaccharose (5 μg). K64-CPS and K47-CPS were purified by standard methods from K. pneumoniae strain 2410 and strain 2226, respectively (18, 19). Serially diluted serum samples were added to wells in duplicate. The secondary anti-human IgG diluted 1:8,000 were used for detection and measurement. Absorbances were read at 405 nm. Titers were expressed as the inverse of the serum dilution ratios, which could produce an absorbance value 2.1 times higher than the blank.

### Serum bactericidal activity.

Serum bactericidal activity (SBA) was measured using a modified version of previously reported protocols (48, 49). An overnight culture of K. pneumoniae strains HvKP4 (K47), which was isolated from a patient at the Second Affiliated Hospital of Zhejiang University (50), and 19-89-1 (K64) isolated from a patient at Beijing hospital was used to make a suspension of 10^5^ CFU/mL in sterile PBS. Heat-inactivated (HI) (56°C) serum samples of patients or healthy children diluted in 1:10 (25 μL) was mixed with 12.5 μL of bacterial suspension. After rotating gently for 30 min at 37°C, 12.5 μL baby rabbit complement (or HI complement) and 30 μL Hanks’ balanced salt solution (HBSS) (with Ca^2+^, Mg^2+^) were combined with the mixture to reach a final volume of 80 μL. All mixtures were incubated at 37°C for 1 h before 10 μL of samples from each reaction was spotted on LB plates. The colonies were enumerated the following day.

### Statistical analysis.

Statistical tests were performed with GraphPad Prism 9 for Windows. For two-group comparisons of parametric data, unpaired Welch’s *t* tests were performed. Comparisons made from interstudy heterogeneity was assessed by the Fisher’s exact test. *P* values of < 0.05 were considered statistically significant.

### Ethics statement.

Patients and healthy donors consented under the Ethics Review Committee, Shanghai Children’s Hospital, School of Medicine, Shanghai Jiao Tong University (approval 2022R060-E01). After informing the patients and acquiring consent in written form, strain specimens and whole blood were collected.
