# Supplementary material for: The Molecular Epidemiology of Prevalent Klebsiella pneumoniae Strains and Humoral Antibody Responses against Carbapenem-Resistant K. pneumoniae Infections among Pediatric Patients in Shanghai
Source: mSphere. 2022 Sep 7;7(5):e00271-22. doi: 10.1128/msphere.00271-22 (PMC9599505; doi:10.1128/msphere.00271-22)
Supplement: TABLE S1 [file msphere.00271-22-s0002.docx]

**Supplemental Materials**

**Table S1. Antimicrobial activities test of CRKP strains**

| **Category** | **Antibiotics** | **MIC_90_**  **(μg/ml)** | **Antimicrobial Resistance (%)** | | |
| --- | --- | --- | --- | --- | --- |
|  |  |  | KPC (n=17) | NDM  (n=4) | Total (n=23) |
| Second-generation cephalosporins | FOX | ≥64 | 94.12 | 100 | 95.65 |
|  | CXM | ≥64 | 100 | 100 | 100 |
| T[hird-generation cephalosporins](javascript:;) | CAZ | ≥64 | 94.12 | 100 | 95.65 |
|  | CRO | ≥64 | 94.12 | 100 | 95.65 |
|  | CSL | ≥64 | 100 | 100 | 95.65 |
| Fourth[-generation cephalosporins](javascript:;) | FEP | ≥32 | 100 | 100 | 100 |
| Carbapenems | ETP | ≥8 | 100 | 100 | 100 |
|  | IPM | ≥16 | 100 | 100 | 100 |
|  | MEM | ≥16 | 100 | 100 | 100 |
| Monobactams | ATM | ≥64 | 100 | 100 | 100 |
| Penicillins | TZP | ≥128 | 100 | 100 | 100 |
|  | AM/CA | ≥32 | 94.12 | 100 | 95.65 |
| Aminoglycosides | AMK | ≥64 | 35.29 | 0 | 26.09 |
|  | GEN | ≥16 | 79.47 | 0 | 65.22 |
| Sulfonamides | SXT | ≥320 | 88.23 | 25 | 69.56 |
| Tetracyclines | TGC | 2 | 0 | 0 | 0 |
| Polypeptide antibiotics | COL | 1 | 5.88 | 0 | 4.35 |

CAZ, ceftazidime; CRO, ceftriaxone; FEP, cefepime; ETP, ertapenem; IPM, imipenem; MEM, meropenem; ATM, aztreonam; TZP, piperacillin-tazobactam; AMK, amikacin; GEN, gentamicin; SXT, sulfamethoxazole/trimethoprim; TGC,tigecycline ; CSL, cefoperazone-sulbactam; FOX, cefoxitin; CXM, Cefuroxim; AM/CA, Amoxicillin and Clavulanate Potassium; COL, Colistin.
