# Supplementary material for: The Molecular Epidemiology of Prevalent Klebsiella pneumoniae Strains and Humoral Antibody Responses against Carbapenem-Resistant K. pneumoniae Infections among Pediatric Patients in Shanghai
Source: mSphere. 2022 Sep 7;7(5):e00271-22. doi: 10.1128/msphere.00271-22 (PMC9599505; doi:10.1128/msphere.00271-22)
Supplement: TABLE S2 [file msphere.00271-22-s0003.docx]

**Supplemental Materials**

**Table S2. Resistance genes of CRKP strains**

| **Carbapenemase genes** | **ESBL genes** | **Number of isolates** |
| --- | --- | --- |
| KPC (n=17) | SHV, CTM, TEM | 10 |
|  | CTM, TEM | 6 |
|  | SHV, CTM | 1 |
| NDM (n=4) | SHV, CTM, TEM | 4 |
| IMP (n=1) | SHV, CTM, TEM | 1 |
| OXA-48 & KPC (n=1) | SHV, CTM, TEM | 1 |
