# Supplementary figures and images for: The Molecular Epidemiology of Prevalent Klebsiella pneumoniae Strains and Humoral Antibody Responses against Carbapenem-Resistant K. pneumoniae Infections among Pediatric Patients in Shanghai
Source: mSphere. 2022 Sep 7;7(5):e00271-22. doi: 10.1128/msphere.00271-22 (PMC9599505; doi:10.1128/msphere.00271-22)

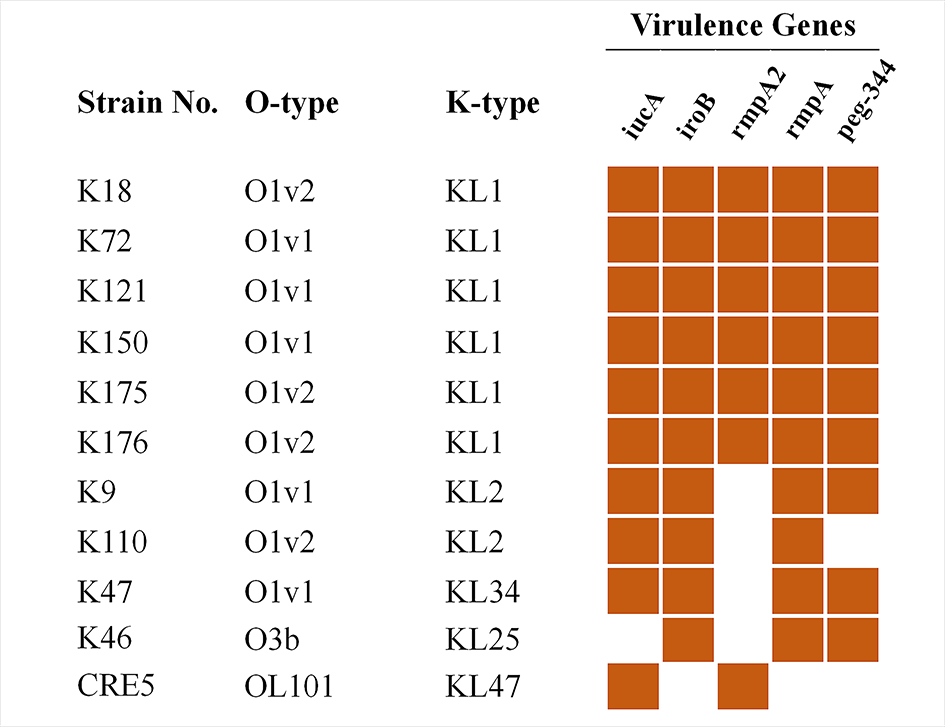

Supplement: FIG S1 [file msphere.00271-22-s0001.tif]
