# Supplementary material for: The Molecular Epidemiology of Prevalent Klebsiella pneumoniae Strains and Humoral Antibody Responses against Carbapenem-Resistant K. pneumoniae Infections among Pediatric Patients in Shanghai
Source: mSphere. 2022 Sep 7;7(5):e00271-22. doi: 10.1128/msphere.00271-22 (PMC9599505; doi:10.1128/msphere.00271-22)
Supplement: TABLE S3 [file msphere.00271-22-s0004.docx]

**Supplemental Materials**

**Table S3. List of primers**

| **Gene** | | **Primer (5’-3’)** | **bp** | | **Annealing Temperature (°C)** |
| --- | --- | --- | --- | --- | --- |
| **hypervirulence-associated genes** | |  |  | |  |
| *iucA* | | F: CCAACTCCGTCCGTACCCTGTCA | 827 | | 55 |
|  | | R: CGAGGGATCGACGATGGTGTTCT |  |  |  |
| *iroB* | | F: GCATTGATCATGTTGCTTGA | 750 | |  |
|  | | R: ATTCTTCAGCGAAGAGATGG |  |  |  |
| *peg-344* | | F: AAAGGACAGAAAGCCAGTG | 528 | |  |
|  | | R: CAATGACGAGGGGGATAATC |  |  |  |
| *rmpA* | | F: ACTGGACTACCTCTGTTTCA | 622 | |  |
|  | | R: GAGCCATCTTTCATCAACCA |  |  |  |
| *rmpA_2_* | | F: CTTTATGTGCAATAAG-GATGTT | 411 | |  |
|  | | R: CTAGGTATTTGATGTGCACC |  |  |  |
| 16S | | F: GGTTCCCCTACGGTTACCTT | 315 | |  |
|  | | R: GCCCTTACGACCAGGGCTAC |  |  |  |
| **Carbapenemase genes** | |  |  | |  |
| *bla*_KPC_ | | F: AGGACTTTGGCGGCTCCAT | 720 | | 55 |
|  | | R: TCCCTCGAGCGCGAGTCTA |  |  |  |
| *bla*_NDM_ | | F: GTTTGGCGATCTGGTTTTC | 621 | | 52 |
|  | | R: CGGAATGGCTCATCACGATC |  |  |  |
| *bla*_OXA-48_ | | F: GCGTGGTTAAGGATGAACAC | 438 | | 52 |
|  | | R: CATCAAGTTCAACCCAACCG |  |  |  |
| *bla*_IMP_ | | F: GGAATAGAGTGGCTTAAYTCTC | 232 | | 55 |
|  | | R: GGTTTAAYAAAACAACCACC |  |  |  |
| *bla*_SPM_ | | F: AAAATCTGGGTACGCAAACG | 271 | | 55 |
|  | | R: ACATTATCCGCTGGAACAGG |  |  |  |
| *bla*_VIM_ | | F: GATGGTGTTTGGTCGCATA | 390 | | 55 |
|  | | R: CGAATGCGCAGCACCAG |  |  |  |
| *bla*_GIM_ | | F: TCGACACACCTTGGTCTGAA | 477 | | 55 |
|  | | R: AACTTCCAACTTTGCCATGC |  |  |  |
| *bla*_AIM_ | | F: CTGAAGGTGTACGGAAACAC | 322 | | 55 |
|  | | R: GTTCGGCCACCTCGAATTG |  |  |  |
| *bla*_SIM_ | | F: TACAAGGGATTCGGCATCG | 570 | | 55 |
|  | | R: AATGGCCTGTTCCCATGTG |  |  |  |
| *bla*_DIM_ | | F: GCTTGTCTTCGCTTGCTAACG | 699 | | 55 |
|  | | R: CGTTCGGCTGGATTGATTTG |  |  |  |
| **ESBL genes** | |  |  | |  |
| *bla*_TEM_ | | F: TCCGCTCATGAGACAATAACC | 296 | | 52 |
|  | | R: ATAATACCGCACCACATAGCAG |  |  |  |
| *bla*_SHV_ | | F: TACCATGAGCGATAACAGCG | 450 | | 52 |
|  | | R: GATTTGCTGATTTCGCTCGG |  |  |  |
| *bla*_CTX-M_ | | F: CGATGTGCAGTACCAGTAA | 585 | | 58 |
|  | | R: TCCGCTCATGAGACAATAACC |  |  |  |
| **AmpC genes** | |  |  | |  |
| MOX | | F: GCTGCTCAAGGAGCACAGGAT | 520 | | 64 |
|  | | R: CACATTGACATAGGTGTGGTGC |  |  |  |
| CIT | | F: TGGCCAGAACTGACAGGCAAA | 462 | |  |
|  | | R: TTTCTCCTGAACGTGGCTGGC |  |  |  |
| DHA | | F: AACTTTCACAGGTGTGCTGGGT | 405 | |  |
|  | | R: CCGTACGCATACTGGCTTTGC |  |  |  |
| ACC | | F: AACAGCCTCAGCAGCCGGTTA | 346 | |  |
|  | | R: TTCGCCGCAATCATCCCTAGC |  |  |  |
| EBC | | F: TCGGTAAAGCCGATGTTGCGG | 302 | |  |
|  | | R: CTTCCACTGCGGCTGCCAGTT |  |  |  |
| FOX | | F: AACATGGGGTATCAGGGAGATG | 190 | |  |
|  | | R: CAAAGCGCGTAACCGGATTGG |  |  |  |
| *wzi* | | F:GTGCCGCGAGCGCTTTCTATCTTGGTATTCC | | 580 | 55 |
|  | R: GAGAGCCACTGGTTCCAGAAYTTSACCGC | | |  |  |
| *wb* O1/O2 | | F1: CGCTATAAGAGCAGCATGCTAG | 1300 | | 56 |
|  | | R1: CGATATCACCTACTGCCAGA |  |  |  |
|  | | F2: TTGTTGAGCCTGACAGGATC | 1600 | |  |
|  | | R2: GCCATTGCTTGCTTGTACAG |  |  |  |
| *wb* O3 | | F1: CTATCGCTACCGTGGCTTTA | 800 | |  |
|  | | R1: TCTCGTCCACAATATCAGCG |  |  |  |
|  | | F2: GCCTACAGTATCTACCTCTG | 900 | |  |
|  | | R2: CGGTAAAGTCAGGATGGAAG |  |  |  |
| *wb* O4 | | F1: CAGAAGCGCGAGTTAATCTG | 700 | |  |
|  | | R1: GGTCCAGTTAGGCTCAATTC |  |  |  |
|  | | F2: GTCAGCGGGAATTATTGGAC | 1200 | |  |
|  | | R2: CTTGAGATCCAGAATGCCAC |  |  |  |
| *wb* O5 | | F1: GCTACCAAACCAGTATGCTG | 1800 | |  |
|  | | R1: AGGTGCGTACTGGAAGTATG |  |  |  |
|  | | F2: GGTGATGAAAGCCAGAATGC | 1400 | |  |
|  | | R2: CAGTGCCTGAAACAGTTTGC |  |  |  |
| *wb* O8 | | F1: CGTGGCAATGGTTTGCTAGT | 1200 | |  |
|  | | R1: TCAATCCACACAACTCGGTC |  |  |  |
|  | | F2: GCTAGTTCGGCAACTAACTCAC | 800 | |  |
|  | | R2: AGTTCCAGCATCGAAGCAACTC |  |  |  |
| *wb* O9 | | F1: CGCGCTCAGTTATTCCATTG | 1000 | |  |
|  | | R1: CTGGCTGATGACAGAGAATC |  |  |  |
|  | | F2: GCATTCCTGTTCGTGTATGG | 900 | |  |
|  | | R2: ATGTCACCGACAGCAAGTAC |  |  |  |
| *wb* O12 | | F1: CTGCAGATGGCTAAACGTGA | 600 | |  |
|  | | R1: CCGTTCGGGCTTGTTCAATA |  |  |  |
|  | | F2: GAAGTCGACTTTGCTGCAGA | 1000 | |  |
|  | | R2: ACGTTGATCAAGCTCCTCTC |  |  |  |
| *wbb* O1 | | F: GATTTCACTTTCCGGGCAAC | 1100 | |  |
|  | | R: GGCTTGCTGAATCACAAGAC |  |  |  |
| *wbb* O2ac | | F: AAACATCGCTGACTCGAGTC | 1000 | |  |
|  | | R: CGACTATGATCGTACCAACG |  |  |  |
| *Wzm* OL101 | | F: GTTAAGAGTATATATACTCACCG | 338 | | 55 |
|  | | R: GATATGACAGATAACGTCTCTAAA |  |  |  |
| *Wzt* OL101 | | F: GAAGCTGTTTCCCCGACGG | 537 | |  |
|  | | R: TCATGATAGTTCATCTCCTTCT |  |  |  |
| O1/O2 variant | |  |  | |  |
| *wbbO*-fw | | TGTTGTGGAGTAAAGGACTGGGCG | v1: 2183  v2: 5020 | | 60 |
| *hisI*-rev | | ACCGCTTCGAGCTGAAGAATGAG |  |  |  |
| O3 subtypes | |  |  | |  |
| *wzm*-fw | | GCGATCTATCGCTACCGTGG | 537 | | 60 |
| *wzm*-rev | | CTGCAGCAGGATATTGACGAAC |  |  |  |
| *wbdD*-O3b | | CAGTACTATCTGCTTCGTCAG | 812 | | 56 |
| *wbdA*-O3b | | GCAAGTTCACGAGCTAGTGTG |  |  |  |
| *wzt*-fw | | CCATCTAAATGGAACCGGGTC | 1227 | | 58 |
| *wzt*-rev | | CTTAAGATCGATGACACCCCAG |  |  |  |
| *wbdA*-fw | | GATTGATGTCCAGGGTTACC | 800 | | 58 |
| *wbdA*-rev | | TCAGGATGCACCTTATACGC |  |  |  |
